# Reply to Sun: Making sense of language change

**DOI:** 10.1073/pnas.2206616119

**Published:** 2022-06-21

**Authors:** Marten Scheffer, Ingrid van de Leemput, Els Weinans, Johan Bollen

**Affiliations:** ^a^Department of Environmental Sciences, Wageningen University, 6700 AA Wageningen, The Netherlands;; ^b^Department of Informatics, Cognitive Science Program, Indiana University, Bloomington, IN 47408

Word use in English books evolved steadily since 1850, until the 1980s when a key component of this gradual change suddenly reversed and surprisingly bounced back to the preindustrial level within a few decades (1). We discovered this using a principal component analysis (PCA) on the long-term dynamics of the 5,000 most-used words. The PCA is not searching for any specific pattern; it merely summarizes the main components of change. This is fundamentally different from the usual approach in historical linguistics, where texts are mined to examine specific hypotheses. For instance, one may show that the meaning of certain words (e.g., *gay*) changed over the past century, or that language is losing markers of power asymmetry (democratization) or becomes less formal (informalization) or speechlike (colloquialization) (2). The results of our PCA are independent of such previous hypotheses. Instead, the PCA may be used to reveal lists of words that most closely follow the hockeystick pattern we found (e.g., *dream*, *angry*, *incredible*) and words that show the opposite pattern (e.g., *statement*, *reference*, *annual*). Such lists invite exploration. What do they tell? What exactly is the nature of the trend reversal that started around 1980? As we show, the PCA trend is closely correlated to sentiment content of texts, but this is not the only element of change. For instance, words on one extreme of the PC axis seem more associated to rationality (*determine* and *conclusion*) while words on the opposite end (such as *feel* or *believe*) seem more related to human experience. At the same time the axis appears to reflect a gradient from a collectivistic (*we*, *they*) to an individualistic (*I*, *he*) focus. Now, Kun Sun suggests that colloquialization of language may better capture our pattern (3). We disagree. While his selection of informal words closely tracks our trend, the frequency of formal words analyzed by Sun started falling already by the end of the Second World War, much earlier than our 1980s trend inflection. We do agree, however, that the thoughtful work on linguistic trends such as colloquialization (3), democratization (2), informalization (4, 5), and conversationalization (6) is invaluable for making sense of patterns such as the one we found. Many changes in the complex network of norms, values, and institutions inevitably happen simultaneously. Language may reflect such multiple intertwined trends, inviting complementary ways of making sense of the patterns. We therefore welcome initiatives such as the one by Sun to mine the open-access treasure trove of word lists that characterize the massive trend reversal in the 1980s exposed by our PCA. Indeed, rather than giving definitive answers our work raises two big questions: How can we best characterize the nature of the component of language that suddenly reverted back to preindustrial levels over the past decades, and what could be the plausible drivers of such change?
